# Supplementary material for: Menopause Averted a Midlife Energetic Crisis With Help From Older Dependent Children and Parents: A Simulation Study
Source: Am J Biol Anthropol. 2026 Jun 28;190(3):e70295. doi: 10.1002/ajpa.70295 (PMC13311743; doi:10.1002/ajpa.70295)
Supplement: Supplementary file 1 — Figure S1: Survival curves. Ache data from Hill and Hurtado (1996). Hadza data from Blurton Jones (2016), !Kung data from Howell (2017). UN values for female e0=35 and male e0=30 from UN life tables (Li and Gerland 2012), using data and code from Gaddy et al. (2025). Figure S2: Weight by sex and age (top) and total energy expenditure (TEE) by age and sex (bottom). Female weight and TEE includes pregnancy increase. Kung weight data from Howell (2009). Ache weight data from Walker et al. (2005). Hadza weight data from Blurton Jones (2016). TEE values from the equation in Bajunaid et al. (2025), except values for age 0–1 from Pontzer et al. (2021). Figure S3: Histogram of the mean differences in age at first marriage for husbands and wives in 177 hunter‐gatherer societies (positive values indicate older ages for men). Note that these values do not necessarily represent the age difference between spouses because young men's and women's first marriages could conceivably have been to much older individuals (which would not be the first marriages for those much older individuals). Data from Marwick et al. (2016). Figure S4: A: Tsimane parental production, children's demands, and net family production. B: Simulated values based on delayed menopause, showing an increase in energy deficits and a decrease in energy surpluses. Figure from Kaplan et al. (2010). Figure S5: Ratio of fertile men (18–60) to fertile women (18–40) by female and male life expectancies at birth in a stationary population. Color gradient and contour lines represent the ratios for different female and male life expectancies. The red dot indicates the life expectancies used in the current study, and the associated ratio of about 1.44 adult men for each fertile woman, which implies male intrasexual competition for mates, a central feature of the grandmother hypothesis. Figure S6: Mean difference in energy balance for each value of each skill ontogeny parameter, relative to its next smallest value, for indiv [file AJPA-190-e70295-s001.pdf]

Supplementary Information: Menopause averted a midlife energetic crisis with help from older dependent children and parents: A simulation study

Edward H. Hagen
Washington State University
edhagen@wsu.edu

1 Hunter-gatherer demography and life history

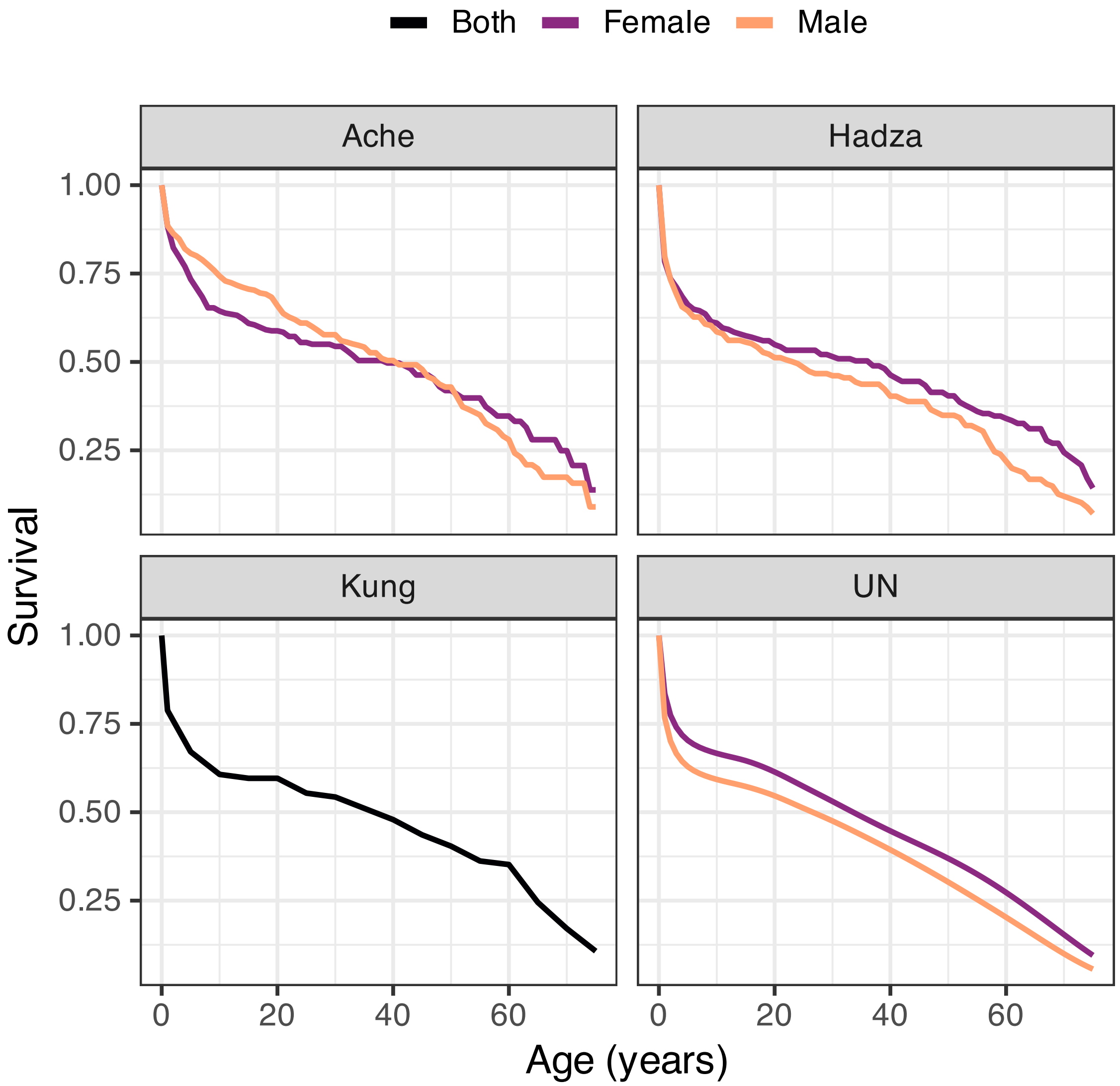

Figure S 1: Survival curves. Ache data from Hill & Hurtado (1996). Hadza data from Blurton Jones (2016), !Kung data from Howell (2017). UN values for female $e_0 = 35$ and male $e_0 = 30$ from UN life tables (Li & Gerland, 2012), using data and code from Gaddy et al. (2025).

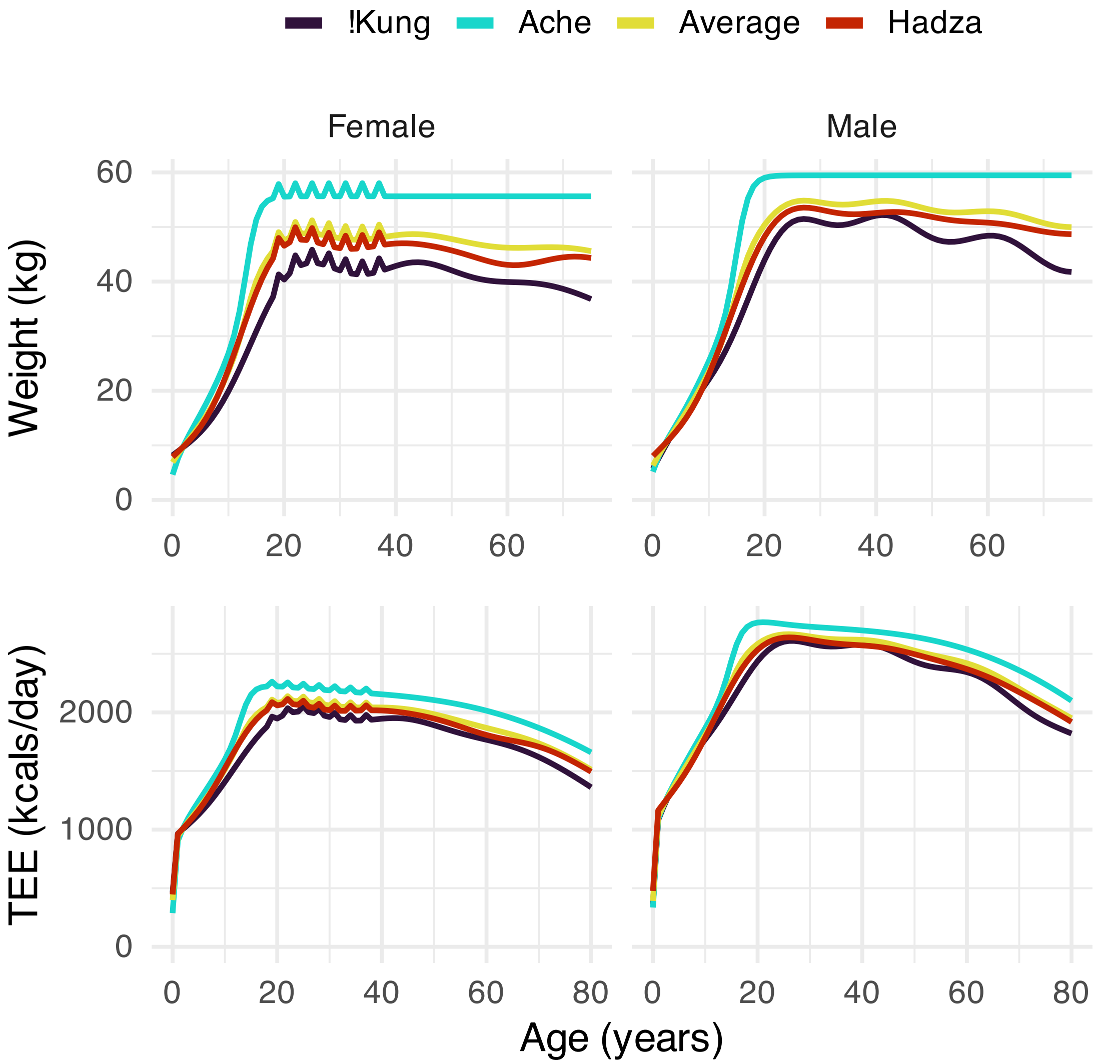

Figure S 2: Weight by sex and age (top) and total energy expenditure (TEE) by age and sex (bottom). Female weight and TEE includes pregnancy increase. Kung weight data from Howell (2009). Ache weight data from Walker et al. (2005). Hadza weight data from Blurton Jones (2016). TEE values from the equation in Bajunaid et al. (2025), except values for age 0-1 from Pontzer et al. (2021).

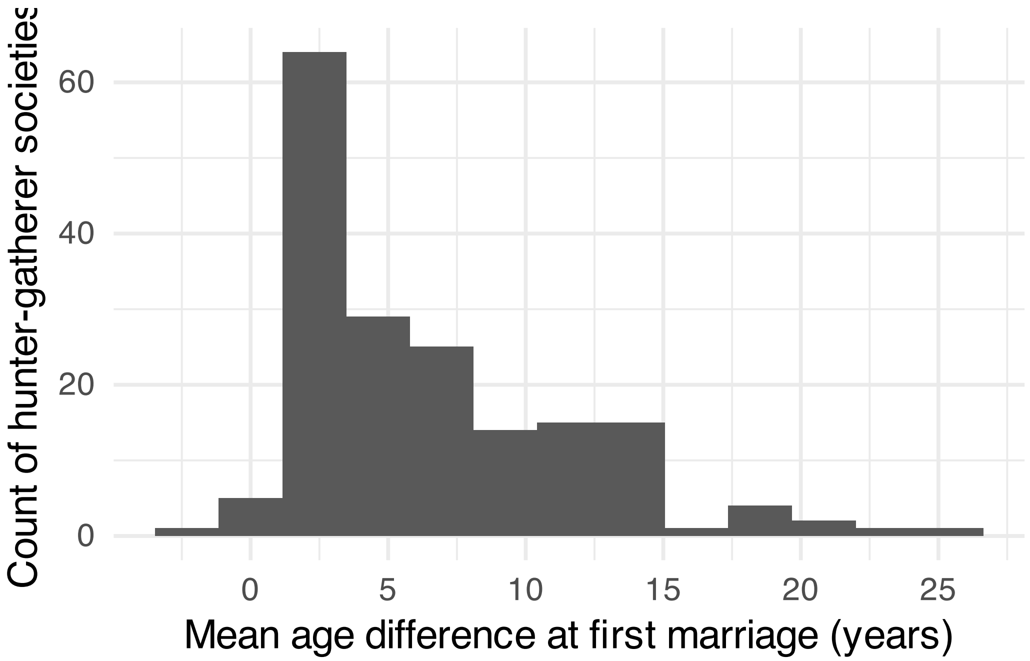

Figure S 3: Histogram of the mean differences in age at first marriage for husbands and wives in 177 hunter-gatherer societies (positive values indicate older ages for men). Note that these values do not necessarily represent the age difference between spouses because young men's and women's first marriages could conceivably have been to much older individuals (which would not be the first marriages for those much older individuals). Data from Marwick et al. (2016).

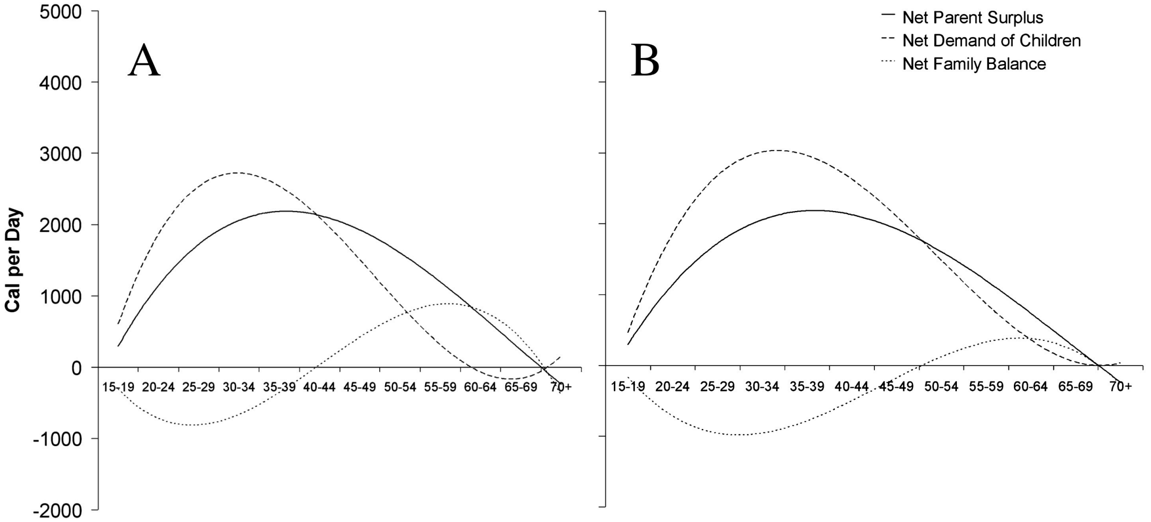

Figure S 4: **A**: Tsimane parental production, children's demands, and net family production. **B**: Simulated values based on delayed menopause, showing an increase in energy deficits and a decrease in energy surpluses. Figure from Kaplan et al. (2010).

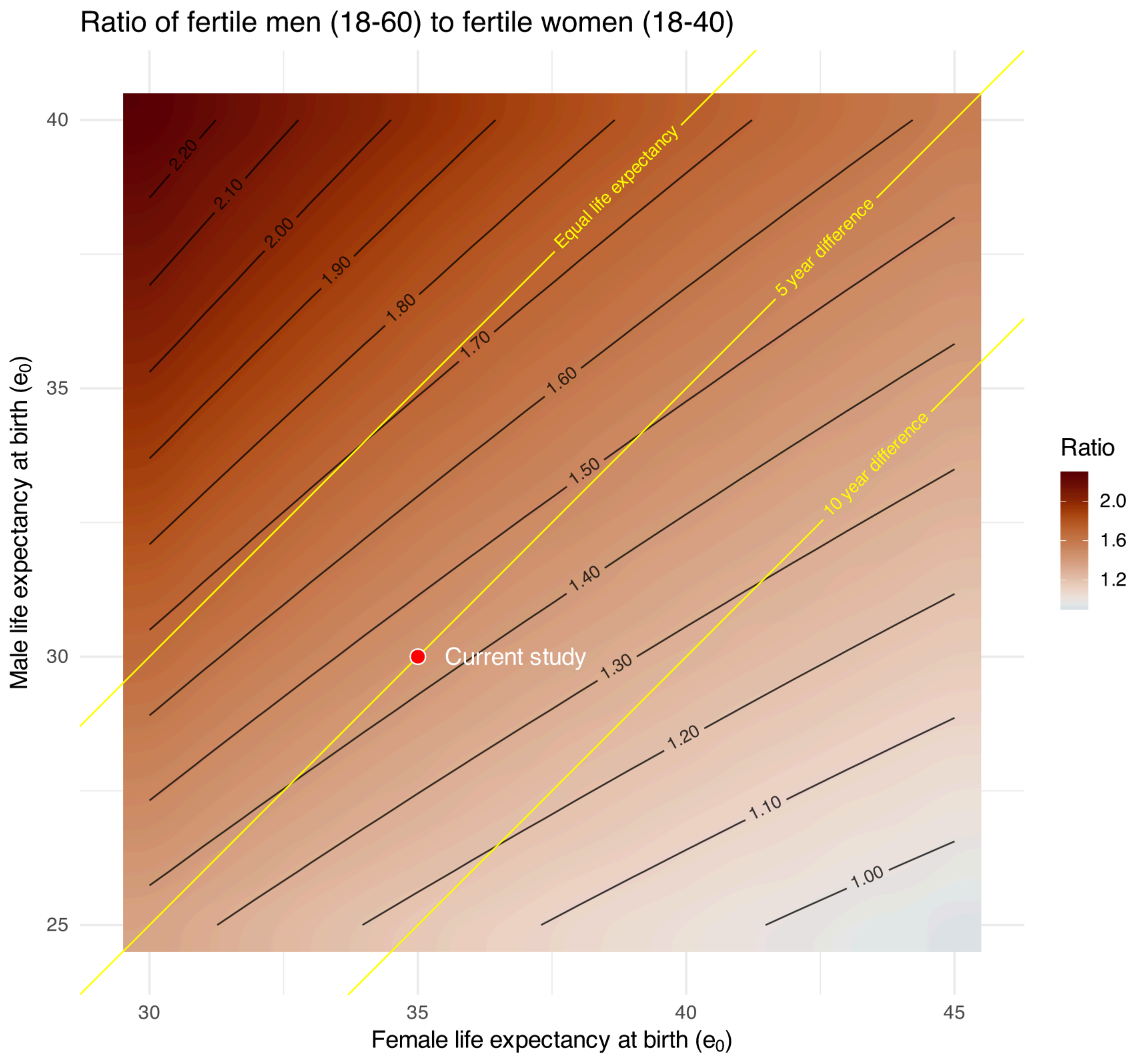

Figure S 5: Ratio of fertile men (18-60) to fertile women (18-40) by female and male life expectancies at birth in a stationary population. Color gradient and contour lines represent the ratios for different female and male life expectancies. The red dot indicates the life expectancies used in the current study, and the associated ratio of about 1.44 adult men for each fertile woman, which implies male intrasexual competition for mates, a central feature of the grandmother hypothesis.

2 Effects of variation in skill ontogeny parameters on energy balance

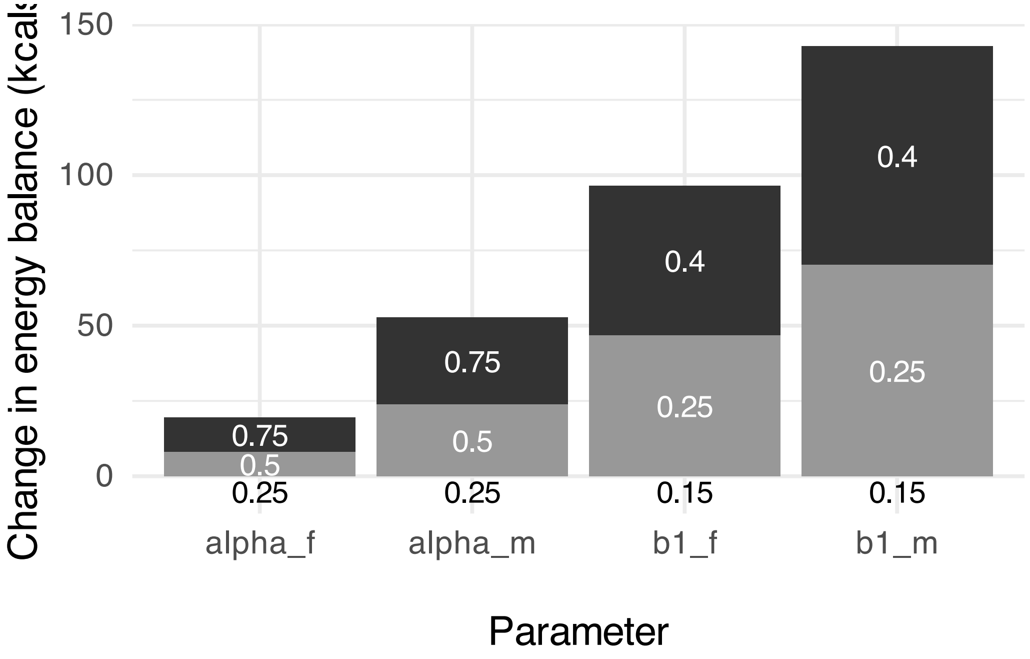

Figure S 6: Mean difference in energy balance for each value of each skill ontogeny parameter, relative to its next smallest value, for individuals ages 0-40 in the population model (averaged over the values of the other parameters). Values on the bars, and at the base of the bars, are the parameter values.

3 Ratio of child production to child consumption

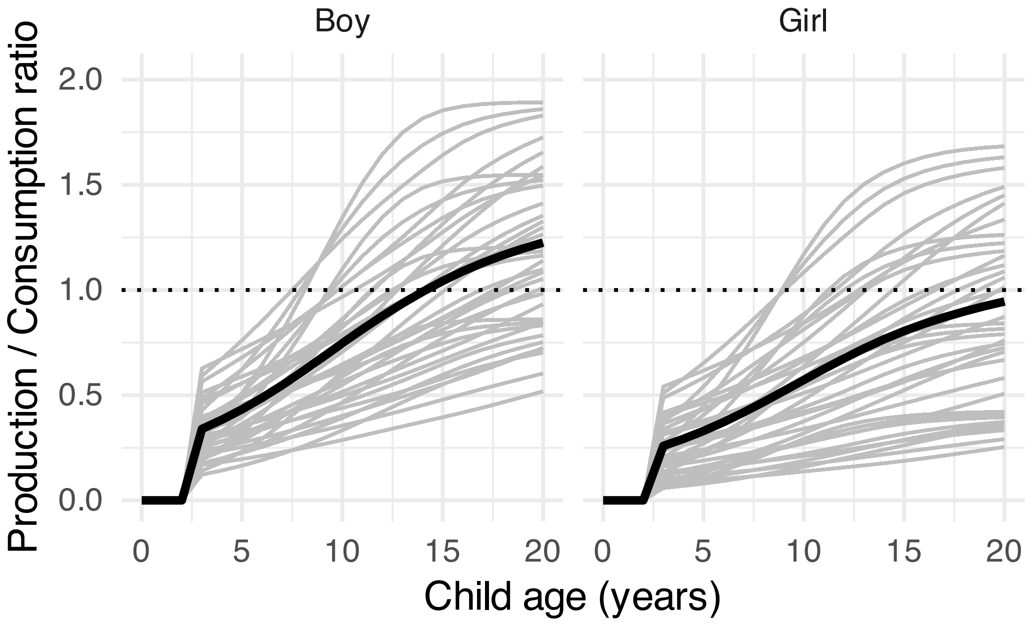

Figure S 7: The ratios of individual children's production to their consumption by age. Grey lines represent trajectories for different combinations of parameter values. Black lines represent the mean ratios averaged across the parameter space.

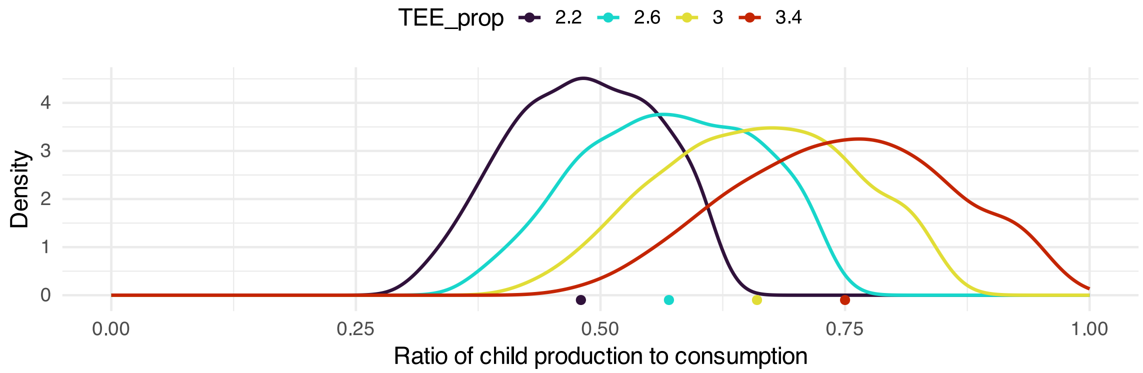

Figure S 8: Distributions of the ratios of the total production of all dependent children (age < 20) to their total consumption, across the parameter space, for different values of TEE_{prop}. Dots along the x-axis are the mean values of each distribution.

4 Effects of variation in ALB and IBI on family composition and energy consumption

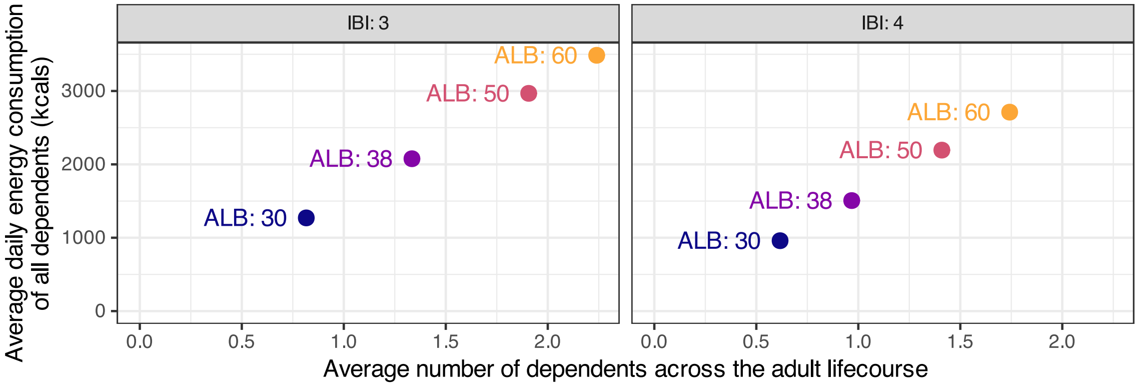

Figure S 9: Average number and daily energy consumption of dependents that families were supporting each day across the adult lifespan, for each value of the ALB and IBI.

5 Energy balance distributions

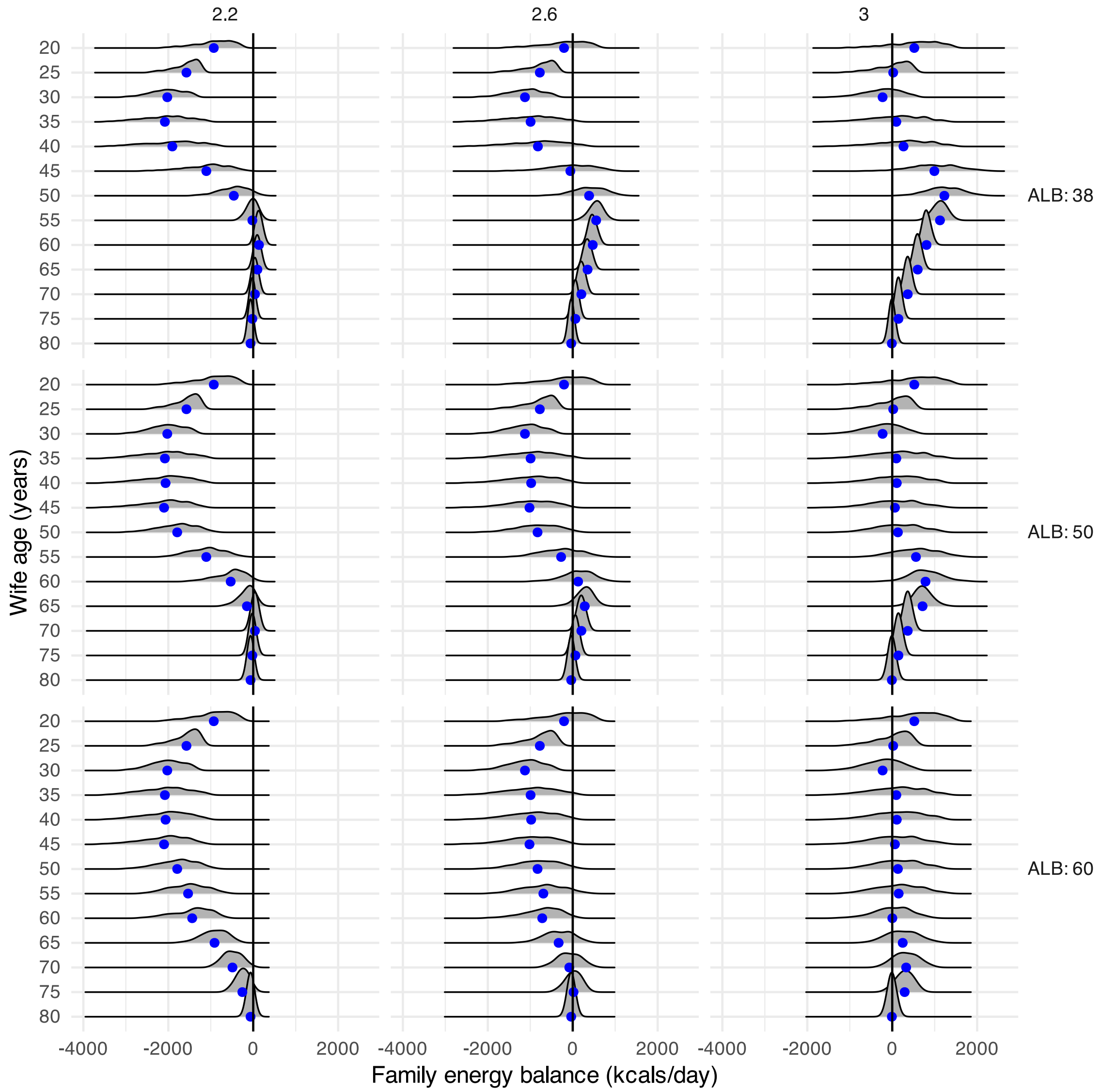

Figure S 10: Variation in energy balance at each age due to variation in skill ontogeny parameters and IBI. Columns: joint adult productivity as a proportion of adult TEE ($TEE_{prop,f} + TEE_{prop,m}$). Rows: ages of last birth (ALB). Blue dots are the mean values. Joint productivity and ALB restricted to intermediate values for clarity.

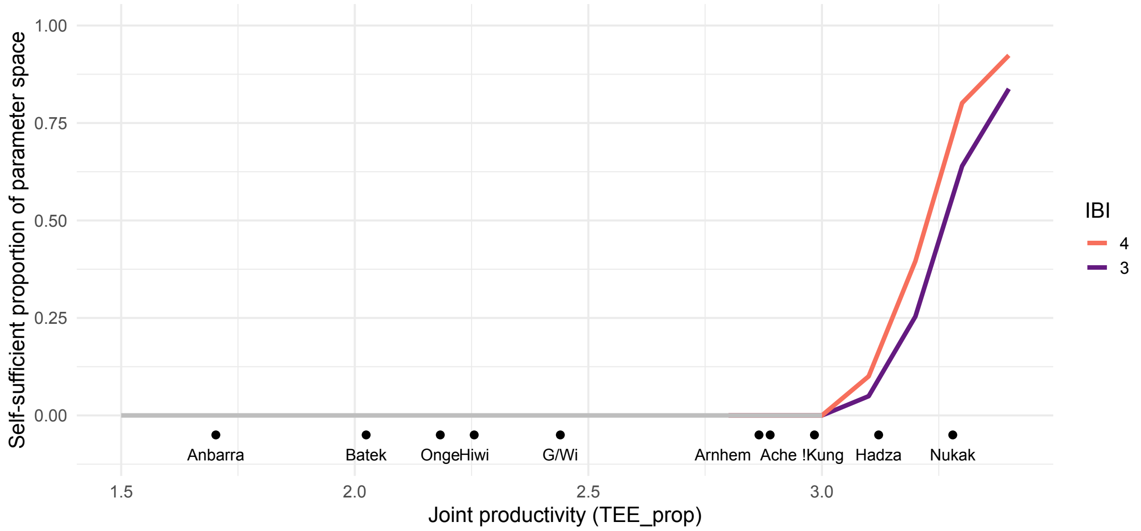

Figure S 11: The proportion of the parameter space in which family energy balance never falls below 0, and families therefore do not need energy transfers from others, as a function of the joint production of the wife and husband. Dots along the x-axis are the joint energy production values from contemporary hunter-gatherers in **?@fig-hg-energy**, for comparison.

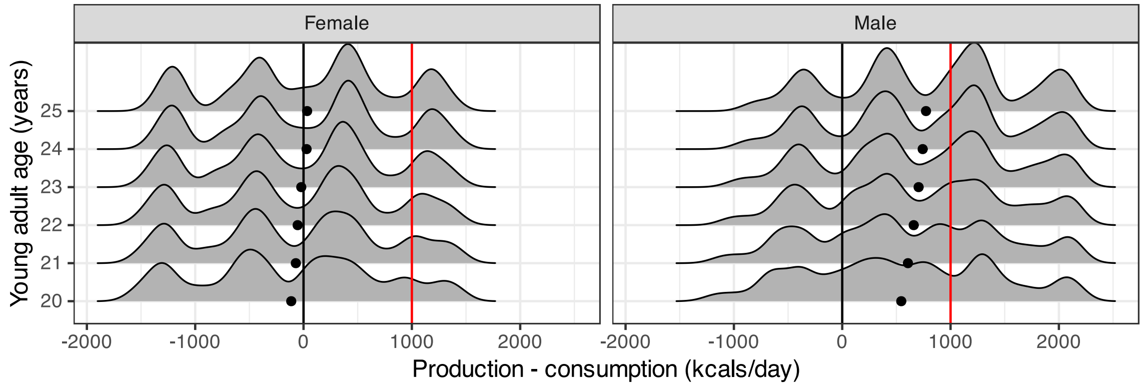

Figure S 12: Variation in net energy production of young adults due to variation in skill ontogeny parameters. The black dots represent the means of each distribution. The vertical red line represents the approximate TEE of an infant or very young child. The four modes are caused by the four sex-specific values of TEE_{prop}. Young adults' positive net energy production could help provision young siblings (helper-at-the-nest), or the siblings of one's spouse, e.g., brideservice.

6 Dependents and surviving parents

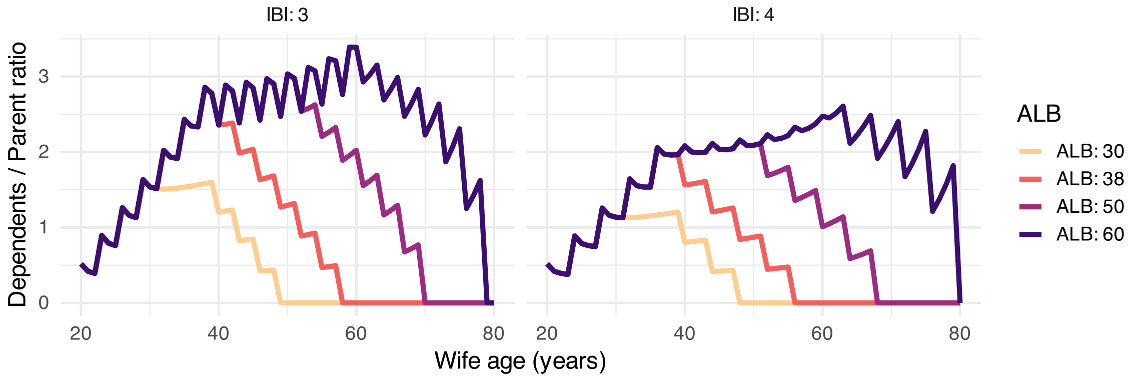

Figure S 13: Ratio of the number of dependents to the number of surviving parents for varying values of ALB, IBI, and wife's age.

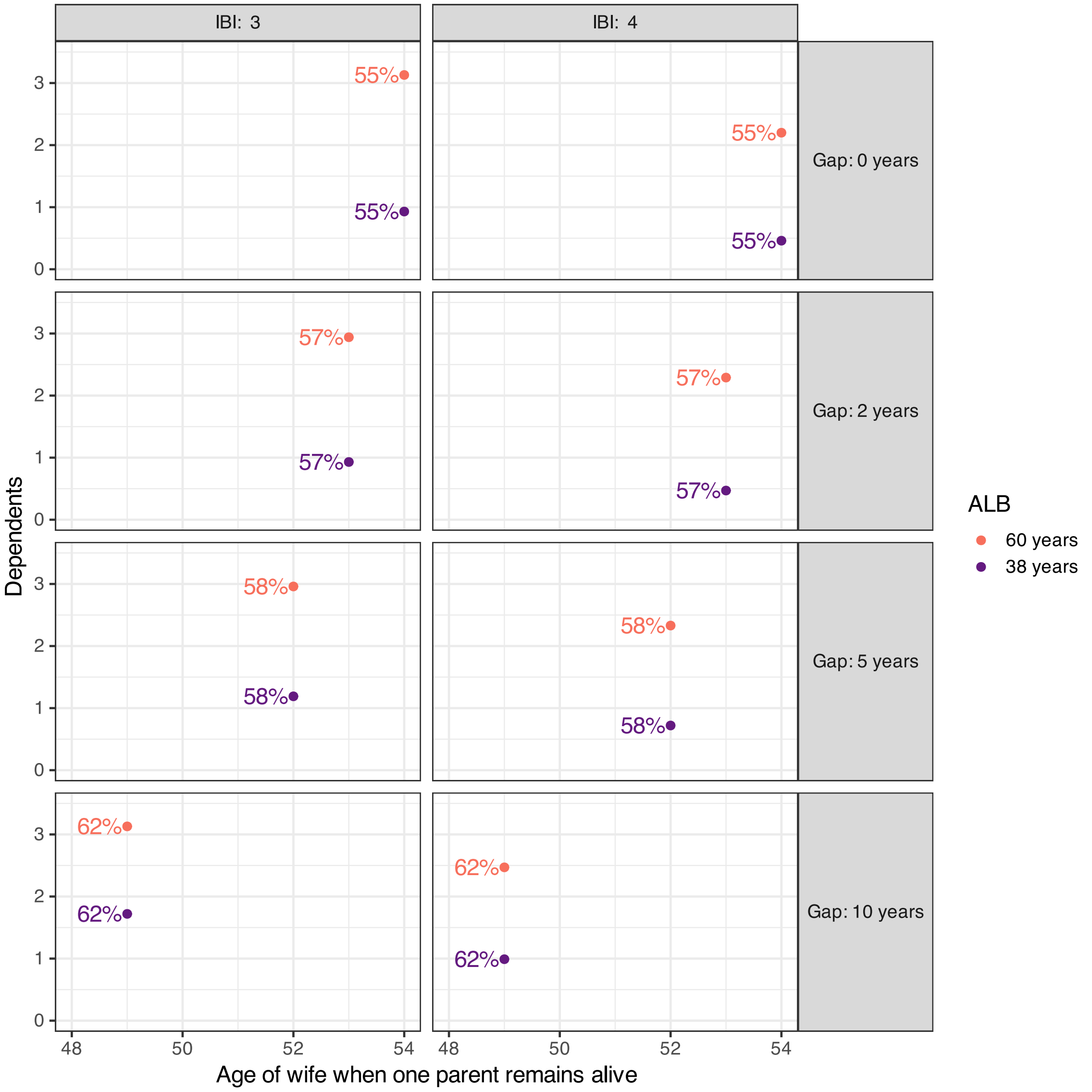

Figure S 14: The number of dependents, on average, at the age when only one parent remains alive due to wife and husband mortality. Gap is the age gap in marriage. Percentage values are the probabilities that the one remaining parent is the wife.

References

Bajunaid, R., Niu, C., Hambly, C., Liu, Z., Yamada, Y., Aleman-Mateo, H., … Speakman, J. R. (2025). Predictive equation derived from 6,497 doubly labelled water measurements enables the detection of erroneous self-reported energy intake. *Nature Food*, *6*(1), 58–71. https://doi.org/10.1038/s43016-024-01089-5

Blurton Jones, N. G. (2016). *Demography and evolutionary ecology of Hadza hunter-gatherers* (Vol. 71). Cambridge University Press.

Gaddy, H., Sear, R., & Fortunato, L. (2025). High rates of polygyny do not lock large proportions of men out of the marriage market. *Proceedings of the National Academy of Sciences*, *122*(40). https://doi.org/10.1073/pnas.2508091122

Hill, K., & Hurtado, A. M. (1996). *Ache life history: The ecology and demography of a foraging people*. Taylor & Francis.

Howell, N. (2009). HTWT observations. Retrieved from http://hdl.handle.net/1807/17996

Howell, N. (2017). *Demography of the Dobe! Kung*. Routledge.

Kaplan, H., Gurven, M., Winking, J., Hooper, P. L., & Stieglitz, J. (2010). Learning, menopause, and the human adaptive complex. *Annals of the New York Academy of Sciences*, *1204*(1), 30–42. https://doi.org/10.1111/j.1749-6632.2010.05528.x

Li, N., & Gerland, P. (2012). Model life tables. Retrieved from https://www.un.org/development/desa/pd/data/model-life-tables

Marwick, B., Johnson, A., White, D., & Eff, E. A. (2016). *Binford: Binford's hunter-gatherer data*. Retrieved from https://CRAN.R-project.org/package=binford

Pontzer, H., Yamada, Y., Sagayama, H., Ainslie, P. N., Andersen, L. F., Anderson, L. J., … Speakman, J. R. (2021). Daily energy expenditure through the human life course. *Science*, *373*(6556), 808–812. https://doi.org/10.1126/science.abe5017

Walker, R., Hill, K., Burger, O., & Hurtado, A. M. (2005). Life in the slow lane revisited: Ontogenetic separation between chimpanzees and humans. *American Journal of Physical Anthropology*, *129*(4), 577–583. https://doi.org/10.1002/ajpa.20306
